# Care Challenges Due to COVID-19 and Mental Health Among Caregivers of U.S. Adults With a Chronic or Disabling Condition

**DOI:** 10.1093/geroni/igab031

**Published:** 2021-10-06

**Authors:** Amanda N Leggett, Alicia Carmichael, Natalie Leonard, Jeannette Jackson, Matthias Kirch, Erica Solway, Jeffrey T Kullgren, Dianne Singer, Preeti N Malani, Richard Gonzalez

**Affiliations:** 1 Department of Psychiatry, School of Medicine, University of Michigan, Ann Arbor, Michigan, USA; 2 Institute for Healthcare Policy and Innovation, University of Michigan, Ann Arbor, Michigan, USA; 3 Institute for Social Research, University of Michigan, Ann Arbor, Michigan, USA

**Keywords:** Aging, Family caregiving, National poll, Pandemic

## Abstract

**Background and Objectives:**

The coronavirus disease 2019 (COVID-19) pandemic poses new challenges for caregivers of adults with chronic or disabling conditions. This study uses nationally representative data to examine the prevalence of pandemic care challenges and supports and their associations with caregiver mental health and interpersonal well-being.

**Research Design and Methods:**

Participants include 311 caregivers aged 50–80 in the United States who were providing care for an adult with a chronic or disabling condition from the June 2020 National Poll on Healthy Aging. Five care challenges (e.g., confusion on public health guidelines) and 2 supports (e.g., physician offered information on care during COVID-19) are treated as predictors of caregiver mental health (care-related stress, self-reported mental health, and depressive symptoms) and interpersonal well-being (interpersonal conflicts, lack of companionship, and isolation).

**Results:**

Each care challenge/support was endorsed by 13%–23% of caregivers. In adjusted models, difficulty getting needed medical care was associated with greater caregiver stress, depressive symptoms, and lower interpersonal well-being. All care challenges universally predicted greater caregiver stress. Caregiving supports were not independently associated with caregiver’ mental health and interpersonal well-being.

**Discussion and Implications:**

Care challenges were associated with caregivers’ mental health and interpersonal well-being during the early months of the pandemic. Some of these challenges may be attributed to changing public health guidelines and practices as the pandemic unfolded, whereas others are relevant to all care contexts (e.g., less support from family). Tools and supports for caregivers must consider both changing policies and care needs.

**Translational Significance:** Using national data, caregivers of adults with chronic or disabling conditions reported on care challenges and supports they experienced during COVID-19. Each challenge and support were reported by less than ¼ of the sample. Yet challenges, particularly difficulty getting needed medical care for the care recipient, were related to negative mental health outcomes for caregivers. Pandemic policies and clinical efforts toward maintaining medical care and social supports for vulnerable older adults may improve patient health, caregiver well-being, and have a great public health benefit. Furthermore, tools and supports for caregivers must consider both changing policies and care needs for maximum impact.

The emergence of a novel coronavirus disease 2019 (COVID-19) caused by severe acute respiratory syndrome coronavirus 2 (SARS-CoV-2) in December 2019 has placed unprecedented strain on interpersonal, health care, and economic systems worldwide. Research on natural disasters suggests that older adults, and especially those with preexisting medical conditions, are particularly vulnerable to global crises (Aldrich, 2012; Behr & Diaz, 2013; Cherniack, 2008; Cloyd & Dyer, 2010). Correspondingly, epidemiological studies demonstrate that older age is related to critical health challenges and mortality associated with COVID-19 (Remuzzi & Remuzzi, 2020). Furthermore, the necessary focus of health care systems on COVID-19 resulted in fewer resources for other medical conditions (Le Couteur et al., 2020; Rimmer, 2020). Thus, adults with chronic or disabling conditions and their family care partners are populations at risk for severe complications from COVID-19, and such complications may act as a barrier to caregiving due to ongoing fear of infection. The aim of this study is to explore the prevalence of pandemic-specific caregiving challenges and supports, such as changing care to reduce risk exposure, and how such challenges or supports are related to caregiver stress and well-being.

Family caregivers remain underrecognized in their role as “frontline” providers helping those with chronic or disabling conditions to age in place and follow public health guidelines. There is pressure on family caregivers to protect their own well-being so they can continue to provide care. Yet a number of social and service-related barriers, due to public health efforts to curb the spread of the virus, may increase care-related stress and reduce well-being for caregivers. Specific policies may lead to restrictions on typical daily activities, reduced physical activity, less social support and instrumental care-related support, loss of outlets (e.g., adult day respite programs, church), increased difficulty in or discouragement from accessing formal care supports and health care services (e.g., in-home nursing services, questioning whether a physician’s visit or elective procedure is necessary), or reliance on these services in new modalities (e.g., telehealth; Mills et al., 2020; Wang et al., 2020).

These issues may be further compounded if the caregiver does not live with the care recipient, forcing caregivers to make decisions about whether to abide by distancing regulations or risk exposure to the care recipient while continuing to provide essential care. Experiences of social isolation and loneliness were common among older adults and caregivers prepandemic and suggest that pandemic restrictions of physical distancing may negatively affect mental and physical health (e.g., increased depression, cognitive decline, and coronary heart disease; Cacioppo et al., 2006, 2010; Chen & Feeley, 2013; Losada-Baltar et al., 2021; Luo et al., 2012; Perissinotto et al., 2012; Rico-Uribe et al., 2018). For example, Savla et al. (2021) found that 59% of dementia caregivers in their sample had sufficient service availability during the pandemic, while the remaining 41% of caregivers had care aides or other services that reduced hours or terminated service. Insufficient support from family and friends was associated with increased role overload experienced by caregivers during the pandemic (Savla, 2021).

Emerging findings suggest that caregiving during the pandemic is associated with increased burden, pain, and psychological distress relative to before the pandemic (Archer, 2021; Sheth et al., 2021). A nationally representative internet panel of U.S. adults examined differences between long-term caregivers (helping for a year or more), short-term caregivers, and noncaregivers in psychological and somatic symptoms (Park, 2021). Relative to noncaregivers, caregivers reported worse fatigue and mental health symptoms and long-term caregivers specifically reported more somatic symptoms (e.g., headaches, abdominal pain). Large surveys from both Germany and the United Kingdom suggested caregiver burden was particularly difficult for those caregivers who usually relied on professional help and had difficulty accessing social services, suggesting the limitation of this resource had a large impact on family caregivers (Budnick et al., 2021; Giebel, 2021). In-depth qualitative interviews with caregivers during the pandemic expound upon challenges caregivers identified which may affect their stress including social isolation, reduced social contacts, care recipient’ health declines, changes in focus to safety and COVID-19 prevention, lack of supports and services, and new caregiving responsibilities (Lightfoot, Moone et al., 2021; Lightfoot, Yun et al., 2021). However, benefits of providing care during a pandemic were also detailed such as care innovations (e.g., enhanced technology, relationship building with the care recipient, and slowing the pace of life and responsibilities). However, examining both potential supports and challenges specific to the pandemic in a national sample and how they independently relate to different facets of caregiver mental health is unknown.

To prevent COVID-19 transmission and maintain caregiver and care recipient health, there is an immediate need to bolster supports for caregivers and understand how pandemic-specific care-related stressors are associated with health and well-being outcomes among older caregivers. Stress Process Theories suggest that the caregiving context, stressors stemming directly from the care recipient’s condition, proliferation of stressors into other areas of the caregiver’s life, and sources of support the caregiver receives to combat stressors all influence the caregiver’s mental health and well-being (Aneshensel, 1995; Pearlin et al., 1990). The goal of this study was to utilize national data on older adults to (a) identify the various health and informal care challenges and supports, specific both to stress process facets of the pandemic caregiving context and supports/lack of supports received, caregivers faced during the COVID-19 pandemic, and (b) how such challenges were associated with the mental health and interpersonal well-being of caregivers. We hypothesize that difficulties related to health care and services will be related to caregiver stress and mental health whereas challenges with receipt of support from family and friends will relate to interpersonal stress.

## Method

The National Poll on Healthy Aging, a recurring nationally representative online cross-sectional survey conducted on various topics, is supported by the University of Michigan (U-M) and the American Association for Retired Persons (AARP). Ipsos conducts the poll by sampling households from its KnowledgePanel, a probability-based web panel designed to be representative of the U.S. population. The data were drawn from the survey conducted between June 3 and 18, 2020 among 2,074 noninstitutionalized adults aged 50–80 years (overall response rate was 78%). Our study selects for those adults who identified as an unpaid caregiver for a relative or friend aged 18 or older with a chronic or disabling condition (*n* = 311). The U-M Institutional Review Board determined the study to be exempt due to deidentified participants.

### Measures

The National Poll on Healthy Aging develops items on timely topics with clinical and/or policy relevance to gain understanding of older adults and caregivers’ perspectives and experiences surrounding topics where limited information exists. The poll format utilizes brief items with simple response scales across multiple domains. The caregiving measures described below stem from this measurement perspective and align with emerging qualitative work on challenges caregivers encountered during the COVID-19 pandemic (Lightfoot, Moone et al., 2021; Lightfoot, Yun et al., 2021).

### Predictors

Participants were surveyed about whether they ever experienced any of the following *challenges or supports related to caregiving during the COVID-19 pandemic* (1 = yes, 0 = no): *Challenges*: Difficulty getting needed in-home and out-of-home services (e.g., nursing, therapy, or respite care), difficulty getting needed medical care for your care recipient, confusion on recommended public health guidelines, providing less care to reduce risk/spread of COVID-19, and decrease in support from family and friends; *Supports*: Increase in support from family and friends and received information from health care professionals about caring for someone with COVID-19. As challenges were formatted as dichotomous items for brevity of the poll, we ask about both an increase and decrease in support in separate items noting that the pandemic may lead to changes in both directions (e.g., increases from neighbors, decreases from family).

### Outcomes

#### Mental health and well-being

Changes in feelings of *caregiving stress during the COVID-19 pandemic* were assessed on a 4-point scale from less stressful to much more stressful. Caregiver self-reported *mental health* was assessed with a self-rating on a 5-point scale from poor to excellent. *Depressive symptoms* over the last 2 weeks were measured with the Patient Health Questionnaire-2 (PHQ-2) that includes little interest or pleasure in doing things and feeling down, depressed, or hopeless measured on a 4-point scale from not at all to nearly every day (Kroenke et al., 2003; Löwe et al., 2005). To examine nuance in associations between caregiving challenges with depressed mood and anhedonia, the PHQ-2 items are considered separately as outcome measures and as a summed score.

#### Interpersonal well-being

Having *interpersonal conflicts* was measured on the same 4-point scale from not at all to nearly every day. Caregivers also reported their feelings of *isolation* and a *lack of companionship* on a scale from 1 (*hardly ever*) to 3 (*often*).

### Controls

Participants provided demographic information including age, gender, race/ethnicity, education, and income.

### Analysis

The first aim was to utilize national data on older adults to identify the frequency of various health and informal care challenges and supports caregivers faced during the COVID-19 pandemic. Descriptive statistics were used to examine sample characteristics and rates of pandemic-related challenges and supports. The total number of challenges participants reported is also described.

Our second aim was to explore how the pandemic challenges and supports were associated with the mental health and interpersonal well-being of caregivers and this was examined in two ways. First, *t*-tests were used to examine mean differences in mental and interpersonal well-being of the caregivers between those who did and did not endorse each care challenge or support. Next, each COVID-19 care challenge or support that exhibited a significant difference between means in three or more mental health and interpersonal well-being outcomes was included as a predictor in an ordinary least squares regression model to estimate unique associations between each care challenge and the mental health and interpersonal well-being outcomes. We used a criterion of at least three significant effects to reduce the number of care challenge and support predictors in the subsequent multiple regressions and evaluated the impact of this criterion through a sensitivity analysis where different criteria were used. If we used a more liberal approach of accepting a predictor with two significant associations, we would have included one additional predictor “difficulty getting needed in-home and out-of-home services.” Whereas, if we were stricter and included a criterion of four significant bivariate associations, we would lose two predictors, “confusion on recommended public health guidelines” and “providing less care to reduce risk/spread of COVID-19.” All regression analyses accounted for controls listed above as main effects to control for intercept differences of these variables and used survey weights to generate nationally representative estimates. Standardized estimates are reported.

## Results

### Sample Characteristics

The majority of caregivers were aged 50–64 (65.0%), female (58.7%), and White (69.3%), with 8.1% of the sample being Black, 15.4% Hispanic, and 7.2% another/multiple racial groups. The majority of caregivers found caregiving to be more stressful during the first 3 months of the pandemic compared to before the pandemic (37.0% a little more, 22.3% much more). Feelings of loneliness were common with 48.7% feeling a lack of companionship and 64.6% feeling isolated at least some of the time. On the other hand, only 6.1% reported interpersonal conflicts over half of the days. Depressive symptoms were relatively high, with 12.5% feeling anhedonia and 8.1% feeling depressed for over half the days. Full sample characteristics are given in Table 1. Between 13.4% and 23.2% of caregivers reported experiencing each care challenge, with 60.8% reporting any of the five challenges. Yet only 23% reported two or more of the five care challenges. The most common care challenges were providing less care to reduce the spread of COVID-19 (23.2%) and experiencing a decrease in support from family and friends (21.3%). Notably, endorsement of the two caregiving supports was also low. Only 14.1% of caregivers reported the support of receiving information from health care professionals about caring for someone with COVID-19, while 17.5% had an increase in support from family and friends. There were four care challenges associated with three or more outcomes in the two-sample *t*-tests and included in subsequent regression models. These were difficulty getting needed medical care for the care recipient, confusion on recommended public health guidelines, providing less care to reduce risk of COVID-19 spread, and decrease in support from family and friends. Among the challenges not included in the regression models, caregivers reporting difficulty getting needed services had greater caregiver stress (*t* = 3.29, *p* < .01) and worse self-rated health (*t* = 2.33, *p* < .05) on average. On the other hand, the two supports were largely unrelated to caregiver mental health and interpersonal well-being, with the exception being that those reporting an increase in support had greater feelings of a lack of companionship (*t* = 2.03, *p* < .05). Full results are given in Table 2. The four care challenges significantly associated with three or more outcomes are subsequently tested in terms of their regression coefficients.

**Table 1.** Caregiver Sample Characteristics (*N* = 311)

Sample characteristic	% [95% CI]
Age (years)	
50–64	65.0 [59.4–70.2]
65–80	35.0 [29.8–40.6]
Female	58.7 [52.5–64.6]
Race/ethnicity	
White, non-Hispanic	69.3 [62.9–75.0]
Black, non-Hispanic	8.1 [5.1–12.7]
Hispanic	15.4 [11.2–20.9]
Other, non-Hispanic	7.2 [4.3–11.7]
Education	
High school of less	43.5 [37.4–49.7]
Some college	22.8 [18.4–27.9]
Bachelor’s degree or higher	33.7 [28.3–39.6]
Annual household income	
Less than $30,000	23.4 [18.1–29.6]
$30,000–$59,999	20.8 [16.5–26.0]
$60,000–$99,999	24.2 [19.5–29.7]
$100,000 or more	31.5 [26.3–37.2]
Lack of companionship	
Hardly ever	51.2 [45.1–57.3]
Some of the time	40.7 [34.9–46.8]
Often	8.0 [5.3–12.1]
Isolation	
Hardly ever	35.4 [29.8–41.5]
Some of the time	50.7 [44.6–56.8]
Often	13.9 [9.9–19.0]
Little interest or pleasure in doing things	
Not at all	59.5 [53.4–65.4]
Several days	28.0 [22.9–33.8]
Over half the days	8.0 [5.1–12.3]
Nearly every day	4.5 [2.5–8.0]
Feeling down, depressed, or hopeless	
Not at all	57.7 [51.5–63.6]
Several days	34.2 [28.6–40.2]
Over half the days	6.9 [4.2–11.3]
Nearly every day	1.2 [0.4–3.7]
PHQ-2	
0	48.5 [42.4–54.6]
1	18.7 [14.2–24.2]
2	19.0 [14.8–24.1]
3	6.0 [3.7–9.7]
4	5.7 [3.2–10.0]
5	0.9 [0.3–2.9]
6	1.1 [0.3–3.7]
Interpersonal conflicts	
Not at all	63.9 [57.8–69.7]
Several days	30.0 [24.6–35.9]
Over half the days	4.5 [2.6–8.0]
Nearly every day	1.6 [0.5–4.9]
Mental health	
Excellent/very good	61.7 [55.5–67.5]
Good	27.0 [21.9–32.7]
Fair/poor	11.3 [7.6–16.5]
Caregiver stress	
Much more stressful	22.3 [17.6–27.8]
A little more stressful	37.0 [31.3–43.2]
About the same	37.8 [32.1–43.9]
Less stressful	2.8 [1.3–6.0]
COVID-19 care challenges and supports	
*COVID-19 care challenges*	
Difficulty getting needed in-home and out-of-home services	13.4 [9.7–18.2]
Difficulty getting needed medical care for your care recipient	18.8 [14.5–23.9]
Confusion on recommended public health guidelines	20.9 [16.4–26.4]
Providing less care to reduce risk/spread of COVID-19	23.2 [18.4–28.7]
Decrease in support from family and friends	21.3 [16.6–26.9]
*COVID-19 care supports*	
Increase in support from family and friends	17.5 [13.3–22.5]
Received information from health care professionals about caring for someone with COVID-19	14.1 [10.3–18.9]
Reported any care challenge	60.8 [54.8–66.6]
Number of care challenges reported by participants	
0	39.2 [33.4–45.2]
1	38.0 [32.2–44.1]
2	14.2 [10.5–19.0]
3	4.7 [2.7–7.9]
4	2.7 [1.4–5.4]
5	1.2 [0.4–4.1]

*Note:* CI = confidence interval; PHQ-2 = Patient Health Questionnaire-2; COVID-19 = coronavirus disease 2019.

**Table 2.** Mean Differences in Caregiver Stress, Mental Health, and Interpersonal Well-Being by Pandemic Care Challenges and Support

Pandemic care challenges and supports	Caregiving stress	Self-rated mental health	Having little interest or pleasure	Feeling down, depressed, hopeless	PHQ-2	Having interpersonal conflicts	Lack of companionship	Isolation
Difficulty getting needed in-home and out-of-home services								
No	2.72 (0.05)	2.25 (0.06)	1.57 (0.06)	1.49 (0.04)	1.06 (0.09)	1.41 (0.05)	1.54 (0.04)	1.76 (0.05)
Yes	3.23 (0.14)	2.69 (0.18)	1.62 (0.16)	1.68 (0.15)	1.30 (0.29)	1.63 (0.12)	1.75 (0.12)	1.91 (0.12)
*t*	3.29**	2.33*	0.30	1.20	0.80	1.70	1.68	1.12
Difficulty getting needed medical care for your care recipient								
No	2.65 (0.06)	2.26 (0.06)	1.46 (0.05)	1.43 (0.04)	0.90 (0.08)	1.36 (0.05)	1.51 (0.04)	1.70 (0.05)
Yes	3.40 (0.08)	2.54 (0.16)	2.05 (0.15)	1.88 (0.13)	1.93 (0.26)	1.78 (0.10)	1.81 (0.09)	2.13 (0.09)
*t*	7.67***	1.59	3.60***	3.35***	3.81***	3.91***	3.02**	4.04***
Confusion on recommended public health guidelines								
No	2.70 (0.06)	2.29 (0.07)	1.52 (0.06)	1.44 (0.04)	0.96 (0.09)	1.38 (0.05)	1.53 (0.04)	1.76 (0.05)
Yes	3.11 (0.10)	2.41 (0.15)	1.77 (0.14)	1.81 (0.12)	1.58 (0.24)	1.66 (0.10)	1.70 (0.09)	1.89 (0.09)
*t*	3.47***	0.75	1.62	2.79**	2.37*	2.47*	1.68	1.32
Providing less care to reduce risk/spread of COVID-19								
No	2.67 (0.06)	2.23 (0.06)	1.55 (0.06)	1.46 (0.05)	1.01 (0.10)	1.41 (0.05)	1.53 (0.04)	1.71 (0.05)
Yes	3.18 (0.09)	2.60 (0.15)	1.65 (0.11)	1.69 (0.11)	1.34 (0.20)	1.54 (0.09)	1.70 (0.09)	2.01 (0.09)
*t*	4.85***	2.34*	0.80	1.87	1.48	1.24	1.67	2.94**
Decrease in support from family and friends								
No	2.68 (0.06)	2.28 (0.07)	1.55 (0.06)	1.47 (0.05)	1.02 (0.09)	1.37 (0.04)	1.52 (0.04)	1.73 (0.05)
Yes	3.17 (0.11)	2.43 (0.14)	1.68 (0.13)	1.68 (0.11)	1.35 (0.21)	1.67 (0.12)	1.75 (0.10)	2.00 (0.08)
*t*	4.05***	0.93	0.94	1.74	1.44	2.34*	2.17*	2.83**
Increase in support from family and friends								
No	2.79 (0.06)	2.30 (0.07)	1.56 (0.06)	1.50 (0.05)	1.06 (0.10)	1.43 (0.05)	1.53 (0.04)	1.76 (0.05)
Yes	2.77 (0.12)	2.38 (0.16)	1.63 (0.11)	1.60 (0.10)	1.23 (0.19)	1.48 (0.11)	1.73 (0.09)	1.92 (0.10)
*t*	−0.21	0.44	0.49	0.93	0.80	0.41	2.03*	1.45
Received information from health care professionals about caring for someone with COVID-19								
No	2.81 (0.06)	2.30 (0.06)	1.61 (0.06)	1.53 (0.05)	1.13 (0.10)	1.45 (0.05)	1.57 (0.04)	1.81 (0.05)
Yes	2.65 (0.13)	2.38 (0.22)	1.39 (0.11)	1.44 (0.11)	0.82 (0.20)	1.36 (0.13)	1.54 (0.11)	1.60 (0.11)
*t*	−1.14	0.35	−1.71	−0.76	−1.38	−0.68	−0.23	−1.82

*Notes: N* = 311; PHQ-2 = Patient Health Questionnaire-2; COVID-19 = coronavirus disease 2019. Linearized standard error in parentheses.

*p < .05, **p < .01, ***p < .001.

### Unique Predictors of Mental Health and Interpersonal Well-Being

Ordinary least squares regression (Table 3) results suggested COVID-19 care challenges were independently associated with caregiver mental and interpersonal well-being even adjusting for the other challenges and controls. Difficulty getting needed medical care (β = 0.30, *SE* = 0.09, *p* < .001), confusion on public health guidelines (β = 0.14, *SE* = 0.11, *p* < .01), providing less care to reduce spread (β = 0.15, *SE* = 0.10, *p* < .01), and experiencing a decrease in support from family and friends (β = 0.21, *SE* = 0.11, *p* < .001) all were independently associated with more caregiving stress during the pandemic. On the other hand, the caregiving challenges differentially predicted mental health symptoms and interpersonal well-being. Caregiver’s self-rated mental health was only significantly associated with providing less care to reduce the risk/spread of COVID-19 (β = 0.19, *SE* = 0.15, *p* < .01). Depressive symptoms, on the other hand, were significantly associated with medical and public health challenges, with having difficulty getting needed medical care consistently associated with both depressive symptoms and the overall PHQ-2 score. However, feeling depressed, alone, was significantly associated with confusion on public health guidelines (β = 0.18, *SE* = 0.11, *p* < .01).

**Table 3.** Associations of Pandemic Care Challenges With Caregivers’ Stress, Mental Health, and Interpersonal Well-Being

Pandemic care challenges	Caregiving stress	Self-rated mental health	Having little interest or pleasure	Feeling down, depressed, hopeless	PHQ-2	Having interpersonal conflicts	Lack of companionship	Isolation
	β (SE)	β (SE)	β (SE)	β (SE)	β (SE)	β (SE)	β (SE)	β (SE)
Difficulty getting needed medical care for your care recipient	0.30 (0.09)***	0.08 (0.16)	0.27 (0.15)***	0.20 (0.11)**	0.26 (0.23)***	0.22 (0.11)***	0.13 (0.10)*	0.21 (0.11)**
Confusion on recommended public health guidelines	0.14 (0.11)**	0.03 (0.15)	0.06 (0.15)	0.18 (0.11)**	0.12 (0.23)	0.12 (0.11)	0.10 (0.09)	0.04 (0.09)
Providing less care to reduce risk/spread of COVID-19	0.15 (0.10)**	0.19 (0.15)**	−0.001 (0.11)	0.10 (0.10)	0.05 (0.19)	0.02 (0.10)	0.07 (0.09)	0.14 (0.10)*
Decrease in support from family and friends	0.21 (0.11)***	0.02 (0.14)	0.05 (0.12)	0.10 (0.11)	0.08 (0.21)	0.16 (0.12)*	0.13 (0.09)*	0.14 (0.09)**
*F*	11.36***	3.09***	3.07***	3.83***	4.54***	4.36***	3.20***	5.46***

*Notes: N* = 311; PHQ-2 = Patient Health Questionnaire-2; COVID-19 = coronavirus disease 2019. Ordinary least squares regression models adjust for age, gender, race/ethnicity, education, and income. Standardized estimates are reported.

*p < .05, **p < .01, ***p < .001.

Both difficulty getting needed medical care (interpersonal conflict: β = 0.22, *SE* = 0.10, *p* < .05; companionship: β = 0.13, *SE* = 0.10, *p* < .05; isolation: β = 0.21, *SE* = 0.11, *p* < .01) and experiencing a decrease in support (interpersonal conflict: β = 0.16, *SE* = 0.12, *p* < .05; companionship: β = 0.13, *SE* = 0.09, *p* < .05; isolation: β = 0.14, *SE* = 0.09, *p* < .01) were consistently associated with reporting all interpersonal stressors. Providing less care to reduce spread of COVID-19 was also independently associated with caregiver’ isolation (β = 0.14, *SE* = 0.10, *p* < .05). Confusion on public health guidelines was unrelated to interpersonal well-being.

## Discussion

In this nationally representative sample of U.S. adults aged 50–80 providing care for an adult with a chronic or disabling condition, we found pandemic-specific care challenges (e.g., confusion on public health guidelines) and supports (e.g., increase in support from family and friends) to be reported by 13%–24% of caregivers and that these challenges and supports were associated with self-reported caregiver stress, depressive symptoms, and interpersonal difficulties. These findings align with emerging research on the increased burden and stress for caregivers during the pandemic (Archer et al., 2021; Budnick et al., 2021; Cohen et al., 2021; Giebel et al., 2021; Park, 2021; Savla et al., 2021; Sheth et al., 2021), yet also depart in key ways. Nearly 60% of our sample reported experiencing more caregiving stress during, as opposed to prior to, the pandemic. Additionally, half the sample reported feeling isolated or a lack of companionship at least some of the time during the pandemic, with depressive symptoms prevalent as well. Yet existing studies on COVID-19 caregiving, while showing decreased psychological well-being, often did not consider correlates of such feelings, which are important areas for bolstering support and enhancing the well-being of family caregivers (Archer et al., 2021; Cohen et al., 2021; Park, 2021; Sheth et al., 2021).

While each specific pandemic-related care challenge was experienced by less than a quarter of caregivers, challenges strongly related to caregiver’s mental health and interpersonal well-being during the COVID-19 pandemic. Individual care challenges had relatively low frequency, yet so did potential care supports, with only 14% of caregivers reporting receipt of information from health care professionals about caring for someone with COVID-19. While it is possible that this information was only being disseminated to those actively caring for someone with COVID-19, older caregivers and their care recipients with chronic illness are at higher risk of COVID-19 complications (e.g., chronic conditions increase the risk for COVID-19 hospitalization; Chang et al., 2021) and this information could be of great value (Remuzzi & Remuzzi, 2020).

Difficulty in accessing needed medical care was associated broadly with caregiving stress and poorer mental health and interpersonal well-being. While health care systems mobilized rapidly to adjust policies and practices during the pandemic, those with ongoing health needs and their caregivers may have felt lost while trying to access needed routine and nonroutine care that was not COVID-specific (Le Couteur et al., 2020; Rimmer, 2020). It is also of interest that confusion on public health guidelines was associated with more interpersonal conflicts, as well as with caregiving stress and depressed mood. This confusion on guidelines may relate to disagreements between caregivers, care receivers, and other members of the care team as to the best approaches for the safe provision of care. In our study, those who experienced a decrease in support from family and friends during COVID-19 were more likely to report interpersonal conflict, isolation, and lack of companionship. The National Academies of Sciences, Engineering, and Medicine (2020) found that among adults aged 50 and older, loneliness and social isolation were associated with increased risk for dementia, heart disease, stroke, depression, anxiety, and even mortality. Thus, efforts to help caregivers compensate for lost support during the pandemic may have a major public health benefit. Social support is an important moderator in Stress Process Models (Lazarus & Folkman, 1984; Pearlin et al., 1990). To test this in a supplementary model, we considered a decrease in support as a moderator of the pandemic care challenges on an increase in caregiving stress, but did not find significance. Thus, pandemic challenges were associated with caregiving stress independent of changes in support, and a decrease in support was independently associated with interpersonal stressors. Further, in *t*-tests, those who endorsed an *increase* in support reported a lack of companionship. While surprising, increases in instrumental support do not necessarily correlate with increased companionship. Increased support could bring about a feeling of dependency in the care dyad and may in fact highlight the disparity between support received for the care recipient and social well-being of the caregiver (Gleason et al., 2008; Thoits, 2011; Warner & Adams, 2016). Furthermore, prior work suggests that a caregiver’s satisfaction with social support received is a more important predictor of psychological well-being than the amount, and thus future work should consider perceptions of pandemic support in addition to changes in the amount of support received (Clay et al., 2008).

While the pandemic-specific challenges and support items developed for the National Poll on Healthy Aging are not an exhaustive list of all challenges or enhanced supports caregivers experienced during the pandemic, they do align with themes from recent in-depth qualitative interviews conducted with family caregivers during the pandemic. Lightfoot et al.’s qualitative work found caregivers placed a new primary focus on safety and prevention of exposure, for example, which changed the caregiving role and responsibilities (Lightfoot, Moone et al., 2021; Lightfoot, Yun et al., 2021). Additionally, as examined in our study, they found less availability of supports and services and reduced social contacts and isolation to be common pandemic care challenges. While Giebel et al. (2021) focused specifically on hours of availability of social services and found that limited ability to access such services was associated with worse mental health during the pandemic, this was only a concern expressed by 13% of our sample. This suggests the importance of assessing caregivers’ perceptions of challenges and supports, in addition to data on more objective availability of such services. For our sample, changes to care practices (e.g., limiting care to reduce risk exposure and less support from family and friends) were reported as more common than complications with service availability, medical care access, and public health guideline confusion.

### Limitations

As this study was cross-sectional, we cannot rule out that those with greater depression and stress viewed the pandemic more negatively and thus endorsed more challenges. Given our sample size, we did not have adequate power to test for moderators of the association between pandemic care stressors and caregiving outcomes as the cell size for different groups (e.g., race, educational attainment) endorsing the care challenge often fell below 15. Future research in larger samples may explore whether factors like gender, age, race, and education moderate associations between pandemic care challenges and caregiver well-being. Additionally, as the primary focus of the National Poll on Healthy Aging was not on caregiving, items on the care recipient such as age, relation to the caregiver, and health condition were not ascertained and thus could not be accounted for within analyses. Of note, as outcome measures were Likert scales, we also ran models as ordinal logistic regressions and identified comparable statistical results.

### Implications

Care challenges, many evident prior to COVID-19 yet accentuated by pandemic circumstances, illuminate how disparities in care supports can affect a caregiver’s stress and interpersonal well-being. Stress Process Models of caregiving emphasize how such factors as program availability and support received relate to how a caregiver appraises their care situation and whether a caregiver experiences negative mental health repercussions (Lazarus & Folkman, 1984; Pearlin et al., 1990). Even still, transitions in caregiving due to changing public health guidelines and fears surrounding virus transmission from care provision are underemphasized and potentially unanticipated in such models. Our findings, alongside additional research into caregiving challenges during COVID-19, may help accelerate the development of evidence-based tools and resources for informal caregivers providing care during pandemic contexts, or shelter-in-place orders, or more broadly, for caregivers who are largely homebound due to their care provision or health conditions (Savla, 2020). Our findings suggest that, like in other COVID-19 caregiving studies, availability of social services was a concern, yet in particular, we find that access to medical care amidst pandemic restrictions was a key concern for caregivers associated with more negative mental health symptoms and less interpersonal well-being. As reducing the risk for the spread of COVID-19 among older adults with chronic or disabling conditions relies heavily on the support of informal caregivers, acknowledging the challenges they face in providing care and enabling supports that bolster their ability to maintain care in varying circumstances may have a great public health benefit.
